# Cancer screening in a middle-aged general population: factors associated with practices and attitudes

**DOI:** 10.1186/1471-2458-9-118

**Published:** 2009-04-29

**Authors:** Stéphane Cullati, Agathe I Charvet-Bérard, Thomas V Perneger

**Affiliations:** 1 Division of Clinical Epidemiology, Geneva University Hospitals, Rue Gabrielle Perret-Gentil 6, CH – 1211 Geneva 14, Switzerland; 2 Geneva Foundation for breast cancer screening, Bd de la Cluse 43, CH – 1205 Geneva, Switzerland

## Abstract

**Background:**

The aim of this study was to identify factors associated with cancer screening practices and with general attitudes toward cancer screening in a general population.

**Methods:**

Mailed survey of 30–60 year old residents of Geneva, Switzerland, that included questions about screening for five cancers (breast, cervix uteri, prostate, colon, skin) in the past 3 years, attitudes toward screening, health care use, preventive behaviours and socio-demographic characteristics. Cancer screening practice was dichotomised as having done at least one screening test in the past 3 years versus none.

**Results:**

The survey response rate was 49.3% (2301/4670). More women than men had had at least one cancer screening test in the past 3 years (83.2% vs 34.5%, p < 0.001). A majority of women had had a cervical smear (76.6%) and a mammography (age 30–49: 35.0%; age 50 and older: 90.3%); and 55.1% of men 50–60 years old had been screened for prostate cancer. Other factors associated with screening included older age, higher income, a doctor visit in the past 6 months, reporting a greater number of preventive behaviours and a positive attitude toward screening. Factors linked with positive attitudes included female gender, higher level of education, gainful employment, higher income, a doctor visit in the past 6 months and a personal history of cancer.

**Conclusion:**

Attitudes play an important role in cancer screening practices among middle-aged adults in the general population, independent of demographic variables (age and sex) that determine in part screening recommendations. Negative attitudes were the most frequent among men and the most socio-economically disadvantaged. The moderate participation rate raises the possibility of selection bias.

## Background

Routine cancer screening has become more frequent in developed societies in the past decades [1-5]. However, disparities within populations remain. Screening is more common among people who are older (an effect partially influenced by screening age recommendations), more educated, white, more affluent, those who do not live alone, and those with a personal and/or family history of cancer [2-4,6-9].

Such differences may relate to problems with access to care, but also to attitudes towards screening. Many adults have a positive attitude toward routine cancer screening [10-12], and many overestimate the benefits of screening [13], be it mammography [14,15], cervical smears [16], or prostate cancer screening [17]. In general, people who have a positive opinion of screening tend to undergo screening more often [8,14,18-23] How attitudes toward screening are distributed in society has not attracted much attention. Most studies about screening attitudes and practices have focussed on specific cancers and specific screening tests, and have not examined a general attitude toward cancer screening. Finally, few studies have examined gender differences in this area.

In this population-based study, we examined past screening practices of five common cancers (breast, cervix uteri, prostate, colon, skin) in middle-aged women and men, and identified factors associated with having had at least one cancer screening test. We also measured general attitudes toward cancer screening, identified risk factors for negative attitudes, and correlated them with reported practices. We did not address cancer screening in high risk groups in this study.

We hypothesized that screening practices would be associated with more positive attitudes and that positive attitudes would explain in part the differences in screening practices between population subgroups.

## Methods

### National context

Currently, in Switzerland, there are no official cancer screening recommendations nor any national cancer screening programs for specific types of cancer in the general population. However, the Swiss Cancer League, a private non-profit foundation, has implemented prevention campaigns for colon cancer (since 2004), breast cancer (since 2000) and skin cancer (since 1988). Moreover, six Swiss cantons – including Geneva – have a public breast cancer screening program for women aged 50 to 69 years. Recommendations regarding cancer screening are issued by the Swiss Cancer League and various medical societies and working groups (See Additional file 1: Appendix 1); most are similar to international guidelines.

The Swiss Law on Health Insurance mandates health insurance coverage for all residents. Individual cancer screening is covered in case of (a) family history of cancer (breast, colorectal, skin), if the test is prescribed by a doctor, or (b) routine examination by a doctor (Papanicolau test for cervical cancer, digital rectal examination for prostate cancer). Examinations by a specialist are reimbursed for early diagnosis of melanoma and prostate specific antigen testing. Patients pay for their health care up to an annual deductible limit (between 300 and 2,500 Swiss Francs); beyond that limit, the co-payment is ten percent of the costs, up to an annual upper limit of 700 Francs. These rules apply to screening as to other health care costs.

### Sample and data collection

We conducted a population-based survey by mail among 30–60 years old residents of the canton of Geneva, Switzerland, in the winter of 2004–2005. The survey was approved by the research ethics committee at Geneva University Hospitals. We used a private survey firm that maintains a list of all residents of the canton (with the exception of diplomatic and United Nations staff), which selected a random sample of 4670 individuals (age limits 30–60, equal numbers of men and women) and mailed out the questionnaires. The respondents returned the anonymous but numbered questionnaires to us, and we communicated the study numbers of returned questionnaires to the survey firm so that reminders would be mailed to non-respondents only. This procedure was implemented so as to maintain the anonymity of the participants, in agreement with the requirements set by the research ethics committee.

### Study variables

We asked participants about ever having undergone screening for each of 5 cancers: breast cancer (for women: mammography, ultrasound), cervical cancer (for women: cervical smear), prostate cancer (for men: digital rectal examination, prostate specific antigen test), colorectal cancer (colonoscopy, faecal occult blood test) and skin cancer (examination by a dermatologist). The same questions were repeated, but limiting the recall period to the past three years. The three-year prevalence was the main variable of interest; the questions about lifetime use were inserted to improve respondent recall. Because we wanted to address cancer screening in general, we computed a binary variable of having done (or not) at least one cancer screening test in the past 3 years.

Attitudes toward cancer screening were measured by a modified 5-item scale of negative attitudes toward screening, or cons [24,25]. This scale was originally constructed for mammography screening, and we adapted it to cancer screening in general (see Additional file 1: Appendix 2). We only used the scale of cons, since previous research has shown that this scale is more strongly related to stages of adoption of screening behaviour than the scale of pros [26]. Responses to the five statements are on a five point Likert scale, from "totally agree" to "totally disagree".

We also probed six health-related behaviours: trying to eat a balanced diet (always*, often*, sometimes, never), exercising for at least 30 minutes (three times per week or more often*, once or twice per week*, less than once a week, never), protecting oneself from direct sunlight (always*, often*, sometimes, never), using a seatbelt when in a car (always*, often*, sometimes, never), having teeth controlled by a dentist (once a year*, less than once a year, never), and smoking (smoker, former smoker*, non-smoker*). Responses marked by an asterisk identify responses what we considered as "positive", to compute the sum of health promoting behaviours.

Finally we obtained data on sex, age, educational level (in 5 categories), gainful employment (yes or no), net monthly household income (in 4 categories), country of birth (Switzerland vs other), having had a doctor visit for a health problem in the past 6 months (yes or no), having been hospitalised in the past 6 months (yes or no), having had a cancer (yes or no), being currently treated for a chronic (> 3 months) health problem (yes or no), and a single item about general health (excellent, very good, good, fair, poor). These items had been validated in previous studies [27,28].

### Statistical Analysis

We compared proportions of respondents who reported one or more screening tests in the past 3 years using chi-square tests. Multivariate modelling was conducted using logistic regression, which yielded odds ratios with 95% of confidence intervals. Predictors were selected if they contributed significantly (p < 0.05) to the model. All tests were two-sided.

Because indications for screening tests differ for men and women, we examined predictive factors separately for these two subpopulations, and compared the odds ratios in a logistic regression model that included as predictors gender, the predictive factor, and an interaction term for gender by predictive factor. A non-significant interaction term indicates that the odds ratio does not differ between men and women. In the multivariate model, we reported separate odds ratios for men and women when significant interactions were observed.

Because we hypothesised that attitudes may constitute an intermediate causal variable between respondent characteristics and screening practice, we built one model without attitudinal variables (the score of cons and reported preventive behaviours), and another that included these variables.

In analyzing the scale of cons, we first established that the scale was uni-dimensional using factor analysis, and verified its internal consistency (Cronbach alpha coefficient). We computed a global score whenever at least 3 of the 5 items were answered, between 0 (lowest possible score of cons) and 100 (highest possible score of cons). We also stratified this score as 0–25, 26–50, 51–75, 76–100. We compared subgroups of respondents using analysis of variance (univariate analysis) and general linear regression models (multivariate analysis). All reported tests were two-tailed at a significance level of 0.05.

## Results

In total 2,368 persons returned the questionnaire (50.7% of 4670), but 13 were eliminated because the respondent was outside the age-range, and 54 skipped the section on screening practices, so that the effective sample size was of 2,301 (49.3%). Respondents who had a history of cancer were included in the analysis.

### Sample description

The respondents included 55.0% women and 45.0% men; their mean age was 44.8 years (standard deviation 9.1 years) (Table 1, first column). Most were born in Switzerland, lived as a couple, were gainfully employed, had monthly incomes greater than 4,000 Swiss francs, had had a doctor visit in the past 6 months, but had not been hospitalised recently nor were they treated for a chronic health problem. Most described their health status as good to excellent, practiced 4 to 6 of the 6 health-related preventive behaviours, and had scores of negative attitudes toward screening (cons) below the mid-point value of 50.

**Table 1:** Sample characteristics and cancer screening practices

		At least one screening test in the past 3 years					
	Sample distribution	All		Women		Men	
	N (%)	%	p-value^1^	%	p-value^1^	%	p-value^1^
Sex			< 0.001				
women	1,266 (55.0)	83.2					
men	1,035 (45.0)	34.5					

Age groups			< 0.001		< 0.001		< 0.001
30–39 years	771 (33.5)	51.5		76.2		13.5	
40–49 years	737 (32.0)	54.5		82.1		27.8	
50–60 years	793 (34.5)	77.0		91.5		59.4	

Education (N = 2270)			0.001		0.057		0.013
elementary school	302 (13.3)	55.6		78.9		23.6	
vocational training	663 (29.2)	57.2		81.2		32.6	
high school diploma	217 (9.6)	62.2		83.3		33.0	
technical school	444 (19.6)	68.5		88.9		41.7	
university	644 (28.4)	62.7		83.0		37.4	

Born in Switzerland (N = 2272)	0.101		0.12		0.16		
yes	1,348 (59.3)	62.6		84.5		36.2	
no	924 (40.7)	59.2		81.2		32.0	

Gainful employment (N = 2252)			0.14		0.97		0.049
yes	1,873 (83.2)	60.5		83.0		35.6	
no	379 (16.8)	64.6		83.1		26.6	

Net monthly household income (N = 2176; Swiss Francs)^a^			0.036		0.005		< 0.001
< = 2'000	104 (4.8)	55.8		75.4		23.1	
2'001–4'000	373 (17.1)	60.3		80.5		21.3	
4'001–8'000	967 (44.4)	58.6		81.5		30.7	
> 8'000	732 (33.6)	65.0		88.8		44.4	

Visited a doctor for a health problem in the past 6 months (N = 2275)			< 0.001		0.007		< 0.001
yes	1469 (64.6)	67.7		85.0		42.5	
no	806 (35.4)	49.1		78.7		23.4	

Hospitalisation in the last 6 months (N = 2277)			0.80		0.11		0.43
yes	171 (7.5)	60.2		77.1		38.7	
no	2,106 (92.5)	61.2		83.5		34.1	

Medical treatment for a chronic health problem (> 3 months) (N = 2274)			< 0.001		0.11		< 0.001
yes	657 (28.9)	69.7		85.6		48.4	
No	1,617 (71.1)	57.6		82.0		29.2	

Personal cancer history (N = 2284)			< 0.001		0.008		0.031
no	2,165 (94.8)	60.2		82.4		33.6	
yes	119 (5.2)	76.5		94.4		48.9	

Global health self evaluation (N = 2274)			0.29		0.19		0.06
excellent	276 (12.1)	56.9		78.6		27.4	
very good	753 (33.1)	60.2		84.0		31.2	
good	1,078 (47.4)	62.8		84.3		37.9	
fair	138 (6.1)	63.0		81.3		41.3	
poor	29 (1.3)	51.7		68.4		20.0	

Scale of cons of screening (N = 2297)			< 0.001		< 0.001		< 0.001
0–25	959 (41.8)	77.6		90.8		49.2	
26–50	758 (33.0)	56.3		78.9		33.6	
51–75	449 (19.5)	40.1		72.6		20.6	
76–100	131 (5.7)	43.5		57.4		31.4	

Number of health related preventive behaviours			< 0.001		0.002		< 0.001
0–3	537 (23.3)	46.2		75.1		26.6	
4	523 (22.7)	60.0		82.0		36.3	
5	687 (29.9)	63.2		84.9		32.2	
6	554 (24.1)	74.7		86.9		49.7	

^a ^In 2004, 1 Swiss Franc was equal to 0.46 GBP.

^1 ^p-value: chi-square test

Non-respondents were younger (44.1 vs 44.8; p = 0.006), more likely to be men (51.7% versus 48.3%; p = 0.022) and similar to respondents in their marital status (54.0% versus 55.0% were married; p = 0.53).

### Cancer screening experience

More women than men had had at least one screening test in the past three years (83.2% vs 34.5%, p < 0.001), but then four tests concerned women and only three concerned men. Women had had more screening tests than men (mean 1.8 tests versus 1.4, p < 0.001). A majority of women had had a cervical smear (76.6%) and a mammography (54.0%); 24.6% of men had had a prostate cancer screening. Of the two non-gender-specific tests, screening for colorectal cancer was more frequent among men (12.2% vs 9.1%, p = 0.016), but women reported more often a skin examination by a dermatologist (19.3% vs 14.0%, p = 0.001).

Recommendations concerning screening for breast, prostate and colon cancer are based on the person's age and sex. The reported practices of screening match these recommendations rather well (Table 2). Among respondents with no screening recommendation (men younger than 50 years), 21.4% had had at least one cancer screening test.

**Table 2:** Type of cancer screening test done in the past 3 years

	Women			Men		
	30–49 years N = 830	50–60 years N = 436		30–49 years N = 678	50–60 years N = 357	
	%	%	p-value	%	%	p-value

Breast cancer (mammography, ultrasound)	35.0	**90.3**	< 0.001	-	-	-
Uterus (Pap test)	**73.4**	**83.2**	< 0.001	-	-	-
Prostate (digital rectal exam., prostate-specific antigen test)	-	-	-	8.7	**55.1**	< 0.001
Colon (colonoscopy, faecal occult blood test)	5.4	**16.8**	< 0.001	5.4	**25.1**	< 0.001
Skin (examination by a dermatologist)	19.2	19.0	0.94	12.7	16.2	0.13
						
At least one test in the past 3 years	78.8	91.5	< 0.001	21.4	59.4	< 0.001
						
Number of recommended screening tests	1	3		0	2	

Note: in **bold **= recommended screening tests

Screening practices differed significantly according to respondent characteristics, globally and within each gender (Table 1, second column). Screening was more frequent among respondents who were older, more educated and more affluent, those who had seen a doctor in the past 6 months for health problem or had a chronic health problem, those who had had a cancer, those with positive attitudes toward screening (lower scores of cons), and those who practiced more health-related preventive behaviours. These patterns were similar in men and women, although the relative differences were often larger among men. The associations differed significantly between women and men for four variables: the odds ratios for screening associated with age, with having seen a doctor in the past 6 months and with being treated for a chronic health problem were lower among women than among men; in contrast, odds ratios associated with the scale of cons were lower among men than among women (details not shown, but the interaction terms were used in multivariate models).

In multivariate analysis, reporting at least one cancer screening test in the past 3 years was associated with female gender, older age, higher income and having visited a doctor for a health problem in the last 6 months (Additional file 1: Appendix 2). When cons of screening and reporting health-related behaviours were introduced into the model, the effect of household income was weakened (the odds ratio for income > 8'000 SFr compared to < = 2'000 SFr went from 3.09 to 1.86), but other associations remained unchanged (Table 3). Attitudes were strongly associated with screening among women, but only weakly among men.

**Table 3:** Multivariate logistic regression of cancer screening practices in the past 3 years

	odds ratio (95% confidence interval)			p-value
Sex				
men		1.0		
women		12.15 (9.49;15.55)		< 0.001

Age groups	women	< 0.001	men	< 0.001
30–39 years	1.0		1.0	
40–49 years	1.64 (1.12;2.39)	0.010	2.35 (1.55;3.58)	< 0.001
50–60 years	3.88 (2.49;6.04)	< 0.001	9.08 (5.97;13.79)	< 0.001

Net monthly income per household				< 0.001
< = 2'000		1.0		
2'001–4'000		0.93 (0.52;1.65)		0.80
4'001–8'000		1.16 (0.67;2.00)		0.60
> 8'000		1.86 (1.06;3.27)		0.031

Visited a doctor for a health problem in the last 6 months				
no		1.0		
yes		1.81 (1.44;2.27)		< 0.001

Number of health behaviours				
0–3		1.0		
4–6		1.37 (1.06;1.77)		0.018

Scale of cons of screening	women	< 0.001	men	< 0.001
0–25	8.56 (4.54;16.13)	< 0.001	1.82 (0.96;3.47)	0.07
26–50	3.74 (2.01;6.96)	< 0.001	1.15 (0.61;2.18)	0.66
51–75	2.30 (1.19;4.44)	0.014	0.53 (0.28;1.03)	0.06
76–100	1.0		1.0	

Note: OR = odds ratio; 95% CI = 95% confidence interval

### Attitudes toward cancer screening

Respondents had a generally favourable attitude toward cancer screening. The majority disagreed (totally and rather) with all five negative statements about screening (see Additional file 1: Appendix 3). Between-item Spearman correlation coefficients ranged from 0.35 to 0.50. The scale's internal consistency coefficient was good (Cronbach α = 0.79). The mean score of cons of screening was 35.4 (standard deviation 24.9). The pattern of mean scores of cons across subgroups paralleled the pattern of screening practices (Table 4). There were a few exceptions: attitudes differed little between age-groups and between respondents who were and were not treated for a chronic health problem; in contrast better health status was associated with more favourable attitudes toward cancer screening.

**Table 4:** Mean score of cons of cancer screening

	Mean score of cons of cancer screening					
	All		Women		Men	
		p-value^1^		p-value^1^		p-value^1^
Sex		< 0.001				
Women	30.4					
Men	41.5					

Age groups		0.16		0.47		0.35
30–39 years	35.4		31.4		41.4	
40–49 years	36.7		30.4		42.8	
50–60 years	34.3		29.4		40.3	

Education (N = 2270)		< 0.001		< 0.001		< 0.001
elementary school	43.5		40.5		47.6	
vocational training	38.9		32.2		45.8	
high school diploma	36.0		30.8		43.2	
technical school	33.1		29.3		38.0	
University	29.5		24.2		36.0	

Born in Switzerland (N = 2272)		0.021		0.32		0.009
Yes	34.4		29.9		39.8	
No	36.9		31.3		43.8	

Gainful employment (N = 2252)		< 0.001		< 0.001		< 0.001
Yes	34.1		28.8		40.0	
No	41.7		36.9		51.7	

Net monthly household income (N = 2176; Swiss Francs)^a^		< 0.001		< 0.001		< 0.001
< = 2'000	48.1		46.3		51.1	
2'001–4'000	42.4		36.8		53.1	
4'001–8'000	36.3		30.6		43.3	
> 8'000	29.1		22.9		34.5	

Visited a doctor for a health problem in the past 6 months (N = 2275)		< 0.001		< 0.001		0.18
Yes	33.7		28.9		40.7	
No	38.8		34.3		42.7	

Hospitalisation in the last 6 months (N = 2277)		0.512		0.69		0.49
Yes	36.6		31.4		43.2	
No	35.3		30.4		41.3	

Medical treatment for a chronic health problem (> 3 months) (N = 2274)		0.384		0.60		0.81
Yes	34.7		29.9		41.2	
No	35.7		30.7		41.6	

Personal cancer history (N = 2284)		< 0.001		0.001		0.17
No	36.0		31.1		41.8	
Yes	27.3		21.0		36.9	

Global health self evaluation (N = 2274)		0.002		0.051		0.049
Excellent	36.0		31.9		41.6	
very good	33.8		28.8		39.9	
Good	35.4		30.2		41.5	
Fair	43.0		37.9		49.1	
Poor	39.5		33.7		50.5	

Number of health related preventive behaviours		< 0.001		< 0.001		< 0.001
0–3	42.9		35.3		48.0	
4	37.3		32.9		42.0	
5	32.8		29.7		37.2	
6	29.8		26.6		36.4	

Number of type of test screening in the past three years		< 0.001		< 0.001		< 0.001
0	44.9		44.2		45.1	
1	36.5		36.0		37.4	
2	26.1		24.6		33.0	
3	17.8		17.3		21.1	
4	17.0		17.0		-	

^a ^In 2004, 1 Swiss Franc was equal to 0.46 GBP.

^1 ^p-value: one-way ANOVA

General linear regression modelling showed that the score of cons was higher among men, the less educated, those without employment, the less affluent, those who did not have a doctor visit for a health problem in the past 6 months and those who had not had a cancer (Table 5). Potential interaction terms with sex were not significant.

**Table 5:** Multivariate model of score of cons

	Difference in cons	95% CI		p-value
Sex				< 0.001
Women	ref			
Men	11.9	9.8	13.9	

Education				< 0.001
elementary school	7.1	3.5	10.8	< 0.001
vocational training	5.3	2.5	8.0	< 0.001
high school diploma	3.8	0.0	7.6	0.048
technical school	1.6	-1.3	4.5	0.283
university	ref			

Gainful employment				0.001
no	4.9	2.1	7.8	
yes	ref			

Net monthly income per household (Swiss Francs)				< 0.001
< = 2'000	17.3	12.1	22.5	< 0.001
2'001–4'000	12.0	8.8	15.2	< 0.001
4'001–8'000	6.5	4.1	8.8	< 0.001
> 8'000	ref			

Visited a doctor for a health problem in the last 6 months				< 0.001
no	3.7	1.6	5.8	
yes	ref			

Personal cancer history				0.001
no	7.4	2.9	12.0	
yes	ref			

^a ^In 2004, 1 Swiss Franc was equal to 0.46 GBP.

^1 ^p-value: one-way ANOVA

## Discussion

### Screening practice

Four sets of factors were associated with screening practice in the general adult population: socio-demographic variables (sex, age groups), household income, recent contact with a doctor, and attitudes toward prevention and screening (scale of cons and number of preventive health behaviours).

Screening recommendations vary with age and sex. Screening is recommended from age 50 on for colorectal cancer, breast cancer (in Switzerland; some other countries place that limit at age 40) and for prostate cancer, by some professional societies (e.g., the American Cancer Society [29]). Furthermore, regular screening for cervical cancer is recommended for all sexually active women, whereas no generally recommended screening test exists for men. From this follows that cancer screening should be more frequent among women and among people who are 50 years old or older, and this is indeed what we observed. Thus medical recommendations about cancer screening are implemented in this population. This does not imply that other factors do not play a role. People over 50 years old and women may have different (more positive) attitudes toward cancer screening than younger people and men for a variety of reasons [30]. On the other hand, cancer screening in absence of recommendations is fairly common. It was documented in this study, as well as elsewhere [31]. Such practices may be explained by various misconceptions about screening tests [14,15].

Wealthier respondents were more likely to undergo cancer screening than the poor. This has been documented previously [3,4,6]. It may be due to financial barriers to screening. However, this explanation is probably incomplete in the context of this study, because most costs of cancer screening tests are reimbursed under the Swiss Law on Health Insurance, and everyone is insured. Furthermore, the association between income and screening practice was much weakened after adjustment for attitudes. This suggests that the effect of income was in good part mediated by attitudes.

Cancer screening was associated with visiting a doctor for a health problem in the past 6 months. This echoes a previous study where contact with a gynaecologist was strongly associated with on-schedule mammography [32]. Two mechanisms may be at play: causation and confounding. Obviously, doctors may recommend or even organise specific screening tests during medical visits for other health problems. Alternatively, people who have a higher propensity to use health services will tend to have more medical visits and at the same time to undergo more screening tests.

We found that attitudes toward screening (measured by the scale of cons of screening) and toward prevention in general (measured by the number of preventive health behaviours) were strongly related to screening practice. A correlation between positive attitude and behaviour is consistent with most health psychology models. Similar associations between attitudes toward cancer screening and screening practices have been reported by others [19-22,26]. We extend these findings by assessing this relationship in a population-based study and by taking into account five different cancer screening practices.

Somewhat to our surprise, adjustment for attitudes had little influence on other predictors of screening practice, with the exception of income categories. Positive attitudes toward screening explain high screening rates among the wealthy, but not the differences between men and women or between age-groups.

### Attitudes toward screening

Globally, most respondents had positive attitudes toward cancer screening – a result which has been observed elsewhere [10,12]. Negative attitudes were more common among men, respondents in lower socio-economic strata, those without a recent doctor visit, and those without a personal history of cancer.

The difference in attitudes between women and men was substantial. Women may have a greater familiarity with screening, having been exposed to cervical smear tests on a regular basis, and familiarity may reduce fear or wariness of screening procedures. Similarly, we can only speculate why people of high socio-economic position have more positive attitudes toward cancer screening. It is possible that understanding the benefits of screening requires that individuals project themselves into the future, which may be easier for people of higher social position. This social inequality in attitudes may be important for public screening programmes in their public relations and social marketing activities.

Visiting a doctor for a health problem in the past 6 months and having a history of cancer were associated with more favourable attitudes. People who have experienced cancer have reason to be motivated by preventive activities, including screening [23,33-36]. Attitudes are determined both by external factors, such as social position or access to health care, and by internal factors related to individual health. The coexistence of the two types of determinants of attitudes – the socio-economic and medical environment and the person's own experience – is consistent with several previous studies [11,37].

### Study strengths and limitations

The study design – a population-based survey – was appropriate for the examination of general attitudes toward cancer screening. The large sample size affords good precision of parameter estimates. However, the response rate was moderate at 50%, which raises concerns about selection bias. Furthermore, as with all local or regional studies, whether our findings may be applicable to other populations is uncertain. All self-report questionnaires are susceptible to social desirability bias and to recall bias [38]. A specific concern is that respondents may not have been able to distinguish tests done for screening purposes from medically indicated procedures. Nevertheless, the patterns of associations are consistent with the literature and suggest that a majority of respondents understood the questions as intended. Another limitation of our analysis is its lack of immediate clinical relevance. We studied cancer screening in general, without distinction between the various tests. Test-specific analyses be more clinically meaningful, but would not address our interest in general attitudes and practices. Finally, by asking about screening tests in the past 3 years, we were unable to distinguish between respondents who were or were not on schedule with specific screening tests, as intervals between screening tests vary.

An interesting by-product of this project was the modified Rakowski's scale of cons of screening; this scale was originally built to explore attitudes toward mammography screening [24,26] and its adaptation to general cancer screening did not alter its acceptability or internal consistency. The validity of this scale is supported by its association with reported screening practices. However, this correlation is not perfect; e.g., almost half of the respondents in the highest quartiles of cons had had at least one screening test in the past 3 years. That attitudes and behaviours do not match perfectly is a common finding.

## Conclusion

Attitudes toward prevention and screening play an important role in screening practice. Women and the wealthier respondents looked more favourably upon cancer screening. We believe that public screening programmes should take into account these differences in attitudes, by targeting men and the most socio-economically disadvantaged.

Further work should explore whether attitudes toward screening are associated with repeated cancer screening tests [39]. Finally, future studies should examine what lies behind attitudes toward cancer screening, such as social representations of cancer screening.

## Competing interests

The authors declare that they have no competing interests.

## Authors' contributions

TVP conceived the project, framed the research question and the study design and directed the data analysis and the writing of the manuscript. SC made the literature review, analyzed the data and wrote the manuscript. AICB conceived the project, managed the data collection and critically contributed to the Discussion section. All authors approved the final manuscript.

## Pre-publication history

The pre-publication history for this paper can be accessed here:



## Supplementary Material

Additional file 1 **Appendices 1 to 3**. Appendix 1: Type of cancer screening recommendations for healthy individuals with no risk factors, Switzerland. Appendix 2: Multivariate logistic regression of cancer screening practices in the past 3 years, excluding covariates that reflect attitude toward cancer screening. Appendix 3: Opinions toward cancer screening, among 30–60 year old residents of Geneva, Switzerland, 2004–2005. Click here for file

## References

[B1] Smith RA, Cokkinides V, Eyre HJ (2007). Cancer screening in the United States, 2007: a review of current guidelines, practices, and prospects. CA Cancer J Clin.

[B2] Breen N, Wagener DK, Brown ML, Davis WW, Ballard-Barbash R (1987). Progress in cancer screening over a decade: results of cancer screening from the 1992, and 1998 National Health Interview Surveys. J Natl Cancer Inst.

[B3] Hiatt RA, Klabunde C, Breen N, Swan J, Ballard-Barbash R (2002). Cancer screening practices from National Health Interview Surveys: past, present, and future. J Natl Cancer Inst.

[B4] Swan J, Breen N, Coates RJ, Rimer BK, Lee NC (2003). Progress in cancer screening practices in the United States: results from the 2000 National Health Interview Survey. Cancer.

[B5] Eisinger F, Blay JY, Morere JF, Rixe O, Calazel-Benque A, Cals L, Coscas Y, Dolbeault S, Namer M, Serin D (2008). Cancer screening in France: subjects' and physicians' attitudes. Cancer Causes Control.

[B6] Carlos RC, Fendrick AM, Patterson SK, Bernstein SJ (2005). Associations in breast and colon cancer screening behavior in women. Acad Radiol.

[B7] Duport N, Ancelle-Park R (2006). Do socio-demographic factors influence mammography use of French women? Analysis of a French cross-sectional survey. Eur J Cancer Prev.

[B8] Saraiya M, Hall HI, Thompson T, Hartman A, Glanz K, Rimer B, Rose D (1992). Skin cancer screening among U.S. adults from 1998, and 2000 National Health Interview Surveys. Prev Med.

[B9] Siahpush M, Singh GK (2002). Sociodemographic variations in breast cancer screening behavior among Australian women: results from the 1995 National Health Survey. Prev Med.

[B10] Kamposioras K, Mauri D, Golfinopoulos V, Ferentinos G, Zacharias G, Xilomenos A, Polyzos NP, Bristianou M, Chasioti D, Milousis A (2007). Colorectal cancer screening coverage in Greece. PACMeR 02.01 study collaboration. Int J Colorectal Dis.

[B11] Lewis CL, Kistler CE, Amick HR, Watson LC, Bynum DL, Walter LC, Pignone MP (2006). Older adults' attitudes about continuing cancer screening later in life: a pilot study interviewing residents of two continuing care communities. BMC Geriatr.

[B12] Schwartz LM, Woloshin S, Fowler FJ, Welch HG (2004). Enthusiasm for cancer screening in the United States. JAMA.

[B13] Denberg TD, Wong S, Beattie A (2005). Women's misconceptions about cancer screening: implications for informed decision-making. Patient Educ Couns.

[B14] Chamot E, Perneger TV (2001). Misconceptions about efficacy of mammography screening: a public health dilemma. J Epidemiol Community Health.

[B15] Domenighetti G, D'Avanzo B, Egger M, Berrino F, Perneger TV, Mosconi P, Zwahlen M (2003). Women's perception of the benefits of mammography screening: population-based survey in four countries. Int J Epidemiol.

[B16] Philips Z, Avis M, Whynes DK (2005). Knowledge of cervical cancer and screening among women in east-central England. Int J Gynecol Cancer.

[B17] McFall SL, Hamm RM, Volk RJ (2006). Exploring beliefs about prostate cancer and early detection in men and women of three ethnic groups. Patient Educ Couns.

[B18] Andrykowski MA, Zhang M, Pavlik EJ, Kryscio RJ (2007). Factors associated with return for routine annual screening in an ovarian cancer screening program. Gynecol Oncol.

[B19] Janz NK, Wren PA, Schottenfeld D, Guire KE (2003). Colorectal cancer screening attitudes and behavior: a population-based study. Prev Med.

[B20] Knops-Dullens T, de Vries N, de Vries H (2007). Reasons for non-attendance in cervical cancer screening programmes: an application of the Integrated Model for Behavioural Change. Eur J Cancer Prev.

[B21] Martin RA, Weinstock MA, Risica PM, Smith K, Rakowski W (2007). Factors associated with thorough skin self-examination for the early detection of melanoma. J Eur Acad Dermatol Venereol.

[B22] Merrill RM, Madanat HN (2002). Cervical cancer risk perception and Pap-smear screening. Health Education Journal.

[B23] Straus WL, Mansley EC, Gold KF, Wang Q, Reddy P, Pashos CL (2005). Colorectal cancer screening attitudes and practices in the general population: a risk-adjusted survey. J Public Health Manag Pract.

[B24] Rakowski W, Andersen MR, Stoddard AM, Urban N, Rimer BK, Lane DS, Fox SA, Costanza ME (1997). Confirmatory analysis of opinions regarding the pros and cons of mammography. Health Psychol.

[B25] Rakowski W, Fulton JP, Feldman JP (1993). Women's decision making about mammography: a replication of the relationship between stages of adoption and decisional balance. Health Psychol.

[B26] Chamot E, Charvet AI, Perneger TV Predicting stages of adoption of mammography screening in a general population. Eur J Cancer.

[B27] Cleopas A, Kolly V, Perneger TV (2006). Longer response scales improved the acceptability and performance of the Nottingham Health Profile. J Clin Epidemiol.

[B28] Perneger TV, Chamot E, Bovier PA (2005). Nonresponse bias in a survey of patient perceptions of hospital care. Med Care.

[B29] American Cancer Society Guidelines for the Early Detection of Cancer. http://www.cancer.org/docroot/ped/content/ped_2_3x_acs_cancer_detection_guidelines_36.asp.

[B30] Evans REC, Brotherstone H, Miles A, Wardle J (2005). Gender differences in early detection of cancer. J Mens Health Gend.

[B31] Chamot E, Charvet A, Perneger TV Overuse of mammography during the first round of an organized breast cancer screening program. J Eval Clin Pract.

[B32] Chamot E, Perneger TV (2003). The gynecologist's role in mammography screening in absence of a public health program. Arch Gynecol Obstet.

[B33] Sessa A, Abbate R, Di Giuseppe G, Marinelli P, Angelillo IF (2008). Knowledge, attitudes, and preventive practices about colorectal cancer among adults in an area of Southern Italy. BMC Cancer.

[B34] McCaffery K, Wardle J, Waller J (2003). Knowledge, attitudes, and behavioral intentions in relation to the early detection of colorectal cancer in the United Kingdom. Prev Med.

[B35] Shapiro JA, Seeff LC, Thompson TD, Nadel MR, Klabunde CN, Vernon SW (2008). Colorectal Cancer Test Use from the 2005 National Health Interview Survey. Cancer Epidemiol Biomarkers Prev.

[B36] Janda M, Youl PH, Lowe JB, Elwood M, Ring IT, Aitken JF (2004). Attitudes and intentions in relation to skin checks for early signs of skin cancer. Prev Med.

[B37] Sutton S, Wardle J, Taylor T, McCaffery K, Williamson S, Edwards R, Cuzick J, Hart A, Northover J, Atkin W (2000). Predictors of attendance in the United Kingdom flexible sigmoidoscopy screening trial. J Med Screen.

[B38] Holt K, Franks P, Meldrum S, Fiscella K (2006). Mammography self-report and mammography claims: racial, ethnic, and socioeconomic discrepancies among elderly women. Med Care.

[B39] Chamot E, Charvet AI, Perneger TV (2007). Who gets screened, and where: a comparison of organised and opportunistic mammography screening in Geneva, Switzerland. Eur J Cancer.

