# Supplementary material for: Cancer screening in a middle-aged general population: factors associated with practices and attitudes
Source: BMC Public Health. 2009 Apr 29;9:118. doi: 10.1186/1471-2458-9-118 (PMC2685378; doi:10.1186/1471-2458-9-118)
Supplement: Additional file 1 — Appendices 1 to 3. Appendix 1: Type of cancer screening recommendations for healthy individuals with no risk factors, Switzerland. Appendix 2: Multivariate logistic regression of cancer screening practices in the past 3 years, excluding covariates that reflect attitude toward cancer screening. Appendix 3: Opinions toward cancer screening, among 30–60 year old residents of Geneva, Switzerland, 2004–2005. [file 1471-2458-9-118-S1.doc]

| **Appendix 1**  **Types of cancer screening recommendations for healthy individuals with no risk factors, Switzerland.** | | |
| --- | --- | --- |
|  | Women | Men |
|  |  |  |
| Breast | Mammography every 2 years between ages 50 to 69 ^1^ | --- |
| Cervix uteri | Pap test every 3 years for sexually active women with a cervix, in absence of risk factors and after two consecutive negative tests^2^ | --- |
| Prostate | --- | First test at age 50, then depending on the level of risk established by the first screening^3^ |
| Colon | Women and men 50 years old or older^4^ | |
| Skin | Self-examination 3 to 4 times per year. If doubt, examination by a dermatologist^5^ | |

Sources:

^1^ Swiss Cancer League ([http://www.breastcancer.ch/pdf/fr/Brochure_Le%20depistage_du_cancer_du_sein.pdf](http://www.breastcancer.ch/pdf/fr/Brochure_Le depistage_du_cancer_du_sein.pdf). Date Accessed: July 2008).

^2^ Arbeitsgruppe Guideline Zervixabstrich (40).

^3^ Swiss Society of Urology and Swiss Cancer League (<http://www.swisscancer.ch/fileadmin/swisscancer/praevention/files/ Factsheet_Prostata-f-110607.pdf> Date Accessed: July 2008).

^4^ U.S. Preventive Service Task Force (<http://www.ahrq.gov/clinic/uspstf/uspscolo.htm>. Date Accessed: July 2008).

^5^ Swiss Cancer League (<http://www.hautkrebstag.ch/index.php?id=331> Date Accessed: July 2008).

**Appendix 2**

**Multivariate logistic regression of cancer screening practices in the past 3 years, excluding covariates that reflect attitude toward cancer screening.**

|  | Odd ratio (95% confidence interval) | | | | *P*-value |
| --- | --- | --- | --- | --- | --- |
| Sex |  | |  | |  |
| men | 1.0 | | | |  |
| women | 14.46 (11.37;18.40) | | | | <0.001 |
| Age groups | women | <0.001 | | men | <0.001 |
| 30-39 years | 1.0 |  | | 1.0 |  |
| 40-49 years | 1.58 (1.10; 2.27) | 0.014 | | 2.34 (1.55; 3.53) | <0.001 |
| 50-60 years | 3.60 (2.37; 5.47) | <0.001 | | 8.79 (5.85; 13.21) | <0.001 |
| Net monthly income per household | | |  | | <0.001 |
| <= 2'000 | 1.0 | | | |  |
| 2'001-4'000 | 1.11 (0.63;1.96) | | | | 0.71 |
| 4'001-8'000 | 1.63 (0.96;2.78) | | | | 0.18 |
| > 8'000 | 3.09 (1.79;5.34) | | | | <0.001 |
| Visited a doctor for a health problem in the last 6 months |  | | | |  |
| no | 1.0 | | | |  |
| yes | 1.84 (1.47; 2.29) | | | | <0.001 |

**Appendix 3**

| **Opinions toward cancer screening*, among 30-60 year old residents of Geneva, Switzerland, 2004-2005.** | | | | | |
| --- | --- | --- | --- | --- | --- |
|  | totally agree | rather agree | neither agree nor disagree | rather disagree | totally disagree |
|  | N (%) | N (%) | N (%) | N (%) | N (%) |
| I would probably not have a screening test unless I had a particular health problem (N=2288) | 349 (15.3) | 482 (21.1) | 306 (13.4) | 464 (20.3) | 687 (30.0) |
| When two screening tests in a row are negative, it is unnecessary to have any more (N=2289) | 315 (13.8) | 390 (17.0) | 347 (15.2) | 431 (18.8) | 806 (35.2) |
| If my doctor examines me regularly at his office, it is unnecessary to do screening tests (N=2288) | 194 (8.5) | 419 (18.3) | 390 (17.0) | 559 (24.4) | 726 (31.7) |
| A screening test has a high risk of leading to unnecessary surgery (N=2287) | 92 (4.0) | 107 (4.7) | 493 (21.6) | 542 (23.7) | 1053 (46.0) |
| If my doctor does not mention screening, neither will I (N=2295) | 226 (9.8) | 375 (16.3) | 300 (13.1) | 529 (23.1) | 865 (37.7) |

* Adapted scale of cons of screening (24, 25)
